# Recurrent granular cell tumor of the thyroid: a case report and literature review

**DOI:** 10.1186/s12893-020-00814-8

**Published:** 2020-07-14

**Authors:** Yu-qin He, Hai-zhen Lu, De-zhi Li, Mo-qi Chen, Kai Wang, Zhen-gang Xu, Shao-yan Liu

**Affiliations:** National Cancer Center / National Clinical Research Center for Cancer / Cancer Hospital, Chinese Academy of Medical Sciences and Peking Union Medical College, Beijing, 100021 China

**Keywords:** Thyroid, Granular cell tumor, Recurrent

## Abstract

**Background:**

Granular cell tumor (GCT) of the thyroid is a rare benign tumor of Schwann cell origin with a favorable prognosis and only 10 cases have been reported so far in scientific literature. The present case study describes the first case of recurrent thyroid GCT.

**Case presentation:**

Our case describes a 20-year-old woman who had undergone lobectomy for GCT of the thyroid 4 years ago. Hematoxylin-eosin (HE) staining revealed that the lesion was composed of epithelioid cells with an abundance of eosinophilic granular cytoplasm. Immunohistochemical analysis showed that tumor cells tested positive for S-100 protein and negative for desmin. Both histological and immunohistochemical analyses supported the diagnosis of recurrent GCT of the thyroid.

**Conclusions:**

Our case suggested that a tumor-free margin excision and post-operative follow-up are necessary for the treatment of GCT of the thyroid.

## Background

Granular cell tumor (GCT) is an uncommon type of neoplasm composed of cells in granular cytoplasm, which was described for the first time in 1926 by the Russian pathologist, Aleksei I. Abrikossoff, as ‘granulosa cell myoblastoma’ of a myogenic origin [1]. However, recent immunohistochemical studies have suggested that GCTs are of Schwann cell origin [2]. GCTs are mostly found at the dorsum of the tongue, although they can occur at other anatomical locations, including skin, breasts and internal organs [3]. GCT of the thyroid gland is extremely rare and only 10 cases that have been reported of in scientific literature published in English. All of these 10 cases accepted initial treatment. This case study reports of the first ever case of recurrence of GCTs in the thyroid gland.

## Case presentation

A 20-year-old woman presented with a 2-week history of the presence of a painless mass on the left side of the thyroid bed, on which a lobectomy was performed 4 years before. In 2014, this patient arrived at a local hospital with a painless mass on the left side of the neck. Ultrasound disclosed that there was a nodule, which was approximately 3 cm*2 cm in size in the left lobe of her thyroid. Serum thyroid-stimulating hormone (TSH) levels were normal. Lobectomy was performed to treat the nodule, which was diagnosed as GCT following pathological examination of the postoperative tissue. Immunohistochemical results showed that the lesion tested positive for neuron-specific enolase (NSE), S-100 protein and negative for smooth muscle actin (SMA) and thyroglobulin (Tg). The mitotic index was assessed using Ki-67 staining and was found to be below 1%. There were no postoperative complications, such as hoarseness and hypoparathyroidism that were reported after initial surgery. There was no regular follow-up after the surgery was performed.

**Fig. 1:**
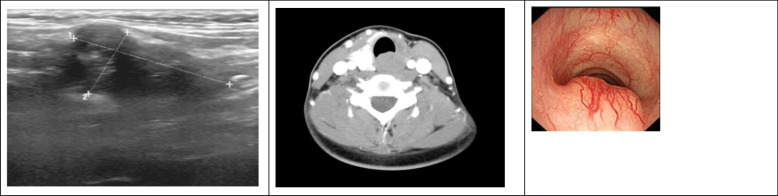
Pre-operative images, endoscopy and FNA performed before recurrent surgery. **a** Ultrasound located a hypoechoic mass on the left thyroid bed; **b** Neck computed tomography scanning showed that an unevenly-enhanced mass located posterior to the fibromuscular membrane of trachea; **c** Bronchoscopy showing that the mass exerted external pressure, causing stenosis of cervical trachea

The patient arrived at our hospital after 4 years, in 2018, for the first time after the initial surgery. This time, ultrasound examination revealed a neoplasm, which was palpable through physical examination, on the left thyroid bed, which was considered as recurrence (Fig. 1a), and multiple solid hypoechoic components of partially cystic nodules and benign lesions were found on the right side of the thyroid. Neck computed tomography scanning with intravenous contrast showed that the mass had a blurred boundary and was slightly unevenly enhanced (Fig. 1b). The laryngoscopy indicated that bilateral vocal cord mobility was normal. The bronchoscopy showed that a posterior subglottic mass, with normal overlying mucosa, compressed the fibromuscular membrane from the outside (Fig. 1c). Moreover, cytology of the fine-needle aspiration (FNA) biopsy together with previous surgical history indicated that the current neoplasm was a recurrent GCT of the thyroid.

To avoid adding an additional superficial scar, the same anterior approach used during the first intervention was used. Intraoperative neuromonitoring was used to identify and protect the recurrent laryngeal nerve (RLN) and the external branch of the superior laryngeal nerve (EBSLN). Intraoperative exploration showed that the recurrent lesion was about 2 cm in diameter and located next to, and also adhered firmly to the left inferior pharyngeal constrictor muscle (upper side of the thyroid) and to the left RLN at the entrance of the larynx. Using a lancet, the tumor was sharply separated from the left RLN which was completely conserved. The left RLN was not bifurcated before it entered the larynx. The function of the RLN and the vagus nerve (in the cervical sheath) were confirmed to be normal using a threshold (100uV) at the beginning and the end of the operation. The tumor was yellowish in appearance, smooth and rubbery to the touch. Part of the tumor was located behind the fibromuscular membrane and was invasive beyond the midline. This patient accepted surgical dissection along with complete thyroidectomy. After reoperation, this patient did not show signs of postoperative dysphonia and had no clinical evidence of hypoparathyroidism.

**Fig. 2:**
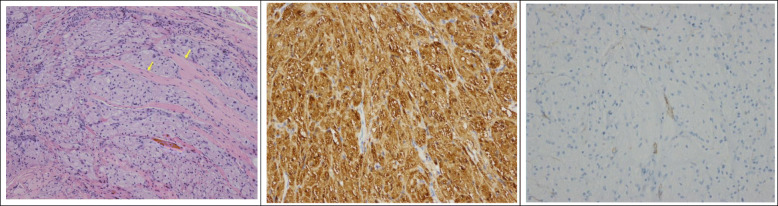
Histopathological examination. **a** Tumor cells consisted of uniformly shaped cells containing an abundance of eosinophilic granular cytoplasm with round or oval nuclei. The yellow arrow indicated tumor tissue infiltration into striated muscle tissue (HE×100); **b** Tumor cells tested strongly positive for S-100 protein. (Immunohistochemical staining, × 200); **c** Tumor cells tested negative for Desmin. (Immunohistochemical staining, × 200)

Histopathological examination using H&E staining revealed that the tumor was composed of epithelioid cells with an abundance of eosinophilic granular cytoplasm. The arrow showed that the tumor tissue infiltrated into striated muscle tissue (Fig. 2a). There was no evidence for nerve invasion in H&E staining. The tumor cells were strongly positive for S-100 protein (Fig. 2b) and negative for Desmin (Fig. 2c). Based on the above findings and the patient’s surgical history, post-operative pathology defined the lesion as recurrent benign GCT of the thyroid. After 6 months of follow-up, no local or distant recurrence was found.

## Discussion and conclusion

GCT, also known as Abrikossoff tumor, is regarded as a neoplasm of neural origin. Although GCT can occur at a wide variety of anatomical locations, the head-and-neck is the most common region, and in particular the tongue. The Head-and-neck region, including the oral cavity, pharynx, larynx, trachea, esophagus, and parotid glands, account for about one-third of all GCTs [4]. GCT is two times more prevalent in females than in males and commonly occurs during the fourth to sixth decades of life [5]. An overwhelming majority of GCT is benign and only 2% is malignant, usually with a poor prognosis [6]. Approximately 60% of malignant GCT patients die of metastasis within 5 years of diagnosis [3].

GCT of the thyroid is so rare that merely 10 cases have been documented in scientific literature published in English. All 10 of these cases were diagnosed as benign GCT, and none resulted in regional or distant metastasis. Nine of these 10 patients were females with an age ranging from 11 to 47 years, with a median of 22 years (Table 1). All patients had undergone initial treatment. Most patients (8/10) had a palpable painless mass in the neck at initial presentation. Ultrasonography on all the patients showed a well-encapsulated mass arising from the thyroid gland, but thyroid function was normal in all these cases. Half of the patients (5/10) attended follow-up visits and no recurrence was found at follow-up.

**Table 1:** Demographics and clinical profiles of cases of GCT of the thyroid reported to date

Author	Year	Sex	Age (year)	Location	Size (cm)	Surgical Method	Positive Expression	Negative Expression	Follow-up (month)
Mahoney et al. [7]	1995	F	12	R	1.5	Lobectomy	S-100	Tg, Vimentin, Ctn, Chromogranin, Epithelial membrane antigen	12
Paproski et al. [8]	2001	F	23	I	1.5	Isthmusectomy	S-100,	Tg	4
Milias et al. [9]	2004	F	43	L	2.5	Total Thyroidectomy	S-100, NSE, Vimentin, Laminin, CD68	Tg, Cytokeratins Cam5.2, AE1\ AE3, Carcinoembryonic antigen, SMA, Ctn	0
Baloch et al. [10]	2005	F	47	L	2.5	Total Thyroidectomy	S-100	Tg, TTF-1, Ctn, Chromogranin	0
Chang et al. [11]	2009	F	12	I	1.55	Isthmusectomy	S-100, Vimentin	Tg, TTF-1, Chromogranin A, Synaptophysin, Ctn, Cytokeratin	15
Espinosa-de-Los-Monteros-Franco et al. [12]	2009	M	21	L	1.8	Total Thyroidectomy	S-100, Calretinin, Protein gene product 9.5	Tg, AE1\ AE3, TTF-1	6
Bowry et al. [13]	2011	F	36	R	0.8	Lobectomy	S-100, CD68, Calretinin, Inhibin A	Tg, AE1\ AE3, Cytokeratin 7, TTF-1, Synaptophysin, Chromogranin A, Ctn	0
Singh et al. [14]	2013	F	11	R	4	Biopsy	S-100		0
Harp et al. [15]	2013	F	27	R	4.23	Rejected surgery	S-100, CD68	Synaptophysin, Chromogranin	0
Chen et al. [16]	2014	F	14	R	3.3	Lobectomy	S-100, CD68, NSE	Tg, TTF-1, Ctn	12

*F* female, *M* male, *R* right, *L* left, *I* isthmus;

Histologically, GCT consists of uniformly shaped large round or polygonal cells, with an abundance of cytoplasm prominently filled with eosinophilic granules [11]. These cells show no mitotic activity, and are positive for S-100, NSE, CD68, laminin, and calretinin, while being negative for Tg, SMA, AE1/AE3, thyroid transcription factor 1 (TTF-1) expression [9, 13]. Positive for S-100 and NSE, as well as negative for SMA are characteristic of Schwann cells, of which they are regarded to originate instead of a striated muscle cell origin. Paproski et al. performed electron microscopy to show that GCT cells contained excess lysosomes as a result of the autodigestion of intracellular myelin [8].

The differential diagnoses included lipoma, medullary thyroid carcinoma (MTC), thyroid follicular epithelial-derived tumor and Hürthle-cell tumor (HCT). Lipoma is usually yellowish in appearance with a capsular shape, similar to GCT. However, HE-staining can clearly differentiate lipoma from GCT through the presence of foamy adipose cells. Although MTC also contains an abundance of cytoplasmic granules, the pre-operative level of serum calcitonin (Ctn) in MTC patients is elevated and MTC cells are positive for Ctn. The most common type of thyroid tumors are thyroid follicular epithelial-derived tumors, including adenomas and differentiated thyroid carcinomas. Due to its origin, MTC is strongly positive for Tg, and its morphology is very similar to that of a normal thyroid gland, which differentiates it from GCT, but not from HCT. HCT tumor cells are also filled with eosinophilic cytoplasm granules, similar to GCT [11]. However, HCT cells are large, round or oval shaped and have well-defined cytoplasm and nuclei with prominent nucleoli. More importantly, HCT cells test positive for Tg, and TTF-1 and negative for S-100, contrary to GCT cells, which are small round or oval with ill-defined cytoplasm, inconspicuous and small nuclei and they test positive for S-100 and NSE and negative for Tg and TTF-1.

The etiology of GCT of the thyroid is unknown but more commonly affects young women. Mahoney reported of GCT in a patient who had a history of receiving high-dose estrogen therapy [7]. However, no association was found between estrogen and progesterone receptor proteins and tumor growth in the patient. Although granular cell changes are associated with trauma, none of these 10 cases had a history of trauma and histological examinations did not reveal any evidence of inflammation, scarring or hemosiderin pigmentation [17].

Surgery is the primary method of treatment for GCT. Neck lymph node dissection and adjuvant radiotherapy are only considered for malignant cases. However, it is difficult to distinguish benign from malignant GCT merely based on histological and immunohistochemical analyses [3]. Malignant GCTs are often accompanied by regional and/or distant metastasis. It is worth mentioning that an infiltrative growth pattern is not an essential factor for the differentiation between benign and malignant GCT. Many cases of benign GCT have been found to show an incomplete tumor capsule and could progressively invade the surrounding tissue, making the recurrence of benign GCT also possible. Our case was the first report on recurrent GCT of the thyroid. We diagnosed our patient as benign GCT because the lesion showed a low nuclear/cytoplasmic ratio and mitotic index, and histologically no necrotic or spindle-shaped cells were found in the tumor.

The recurrence rate for GCT is 8–12%, while this rate increases to 21–50%, allowing for positive surgical margins [18]. Therefore, surgical removal must ensure tumor-free margins. Perusal of all previously reported cases showed that one patient rejected surgical treatment, the extent of excision was not mentioned for another patient who underwent a biopsy, while the other cases had undergone lobectomy or isthmusectomy, and at least three cases had received total thyroidectomy. Our patient had undergone a left lobectomy for the primary lesion, which had been diagnosed as GCT of the thyroid. At that time, residual thyroid was not observed in left side and the lesion was closely attached to left inferior pharyngeal constrictor muscle. The primary GCT may have invaded the muscle resulting in the residual tumor. However, there was not enough evidence to support our conjecture.

In conclusion, GCT of the thyroid is a rare cancer with a good prognosis. So far, all cases of GCT of the thyroid have been reported to be benign tumors with an infiltrative growth pattern. More attention needs to be focused on the association between the tumor and its surrounding tissues, since surgical resection requires a tumor-free margin to avoid recurrence. Regular follow-up after surgery is necessary to monitor treatment outcomes and recurrence. Further understanding on the clinical and histopathological evidence of these rare cases may lead to better diagnosis and the effective treatment of thyroid GCT.

## Data Availability

All data generated or analyzed during this study are included in this article. The data are available from the corresponding author upon a reasonable request.

## Abbreviations

GCT: Granular cell tumor
TSH: Serum thyroid-stimulating hormone
NSE: Neuron-specific enolase
SMA: Smooth muscle actin
Tg: Thyroglobulin
FNA: Fine-needle aspiration
RLN: Recurrent laryngeal nerve
EBSLN: External branch of the superior laryngeal nerve
HE: Hematoxylin-eosin
TTF-1: Thyroid transcription factor 1
MTC: Medullary thyroid carcinoma
HCT: Hürthle-cell tumor
Ctn: Calcitonin
